# Decoction derived from *Allium ascalonicum* L. bulbs and Sojae Semen Praeparatum alleviates wind-cold-type common cold *via* Nrf2/HO-1 pathway and modulation of *Lactobacillus murinus* level

**DOI:** 10.3389/fphar.2024.1364328

**Published:** 2024-05-13

**Authors:** Yuanyuan Jiang, Wenfeng Wei, Jiaxin Zhou, Shixian Qiu, Qixin Yang, Jin hai Huo, Weiming Wang

**Affiliations:** ^1^ School of Traditional Chinese Medicine, Southern Medical University, Guangzhou, China; ^2^ Guangdong Provincial Key Laboratory of Chinese Medicine, Pharmaceutics, Guangzhou, China; ^3^ Guangdong Provincial Engineering Laboratory of Chinese Medicine Preparation Technology Institute of Chinese, Guangzhou, China; ^4^ Heilongjiang Academy of Chinese Medicine Sciences, Harbin, China

**Keywords:** *Allium ascalonicum* L. bulbs, Sojae Semen Praeparatum, wind-cold-type common cold, Nrf2/HO-1 pathway, *Lactobacillus murinus; Glycine max (L.) Merr*

## Abstract

**Background:**

Cong-Chi decoction (CCD) is made using *Allium ascalonicum* L. (shallot) bulbs and Sojae Semen Praeparatum (SSP). Shallot bulbs and SSP are both used regularly in traditional Chinese medicine; however, there are no recent pharmacological studies on their synergistic effects. Despite their roles in the treatment of the common cold for thousands of years, their pharmacological mechanisms of action against wind-cold-type common cold are yet to be explored comprehensively.

**Methods:**

A mouse model was standardized using wind-cold modeling equipment to study the anti-inflammatory, antioxidant, and antiapoptotic effects of CCD. Then, 16S rRNA sequencing was employed to analyze the association between *Lactobacillus murinus* and changes in body temperature. Additionally, the antipyretic effects of *L. murinus* were validated via animal experiments.

**Results:**

The results indicate that CCD improves the symptoms of wind-cold by reducing fever, levels of pro-inflammatory factors, and cellular apoptosis, as well as increasing the blood leukocyte and lymphocyte counts, thereby alleviating lung tissue damage. The effects of CCD are mediated by upregulation of pulmonary Nrf2 and HO-1 expressions, thereby reducing oxidative damage in the lungs, in addition to other anti-inflammatory mechanisms. Furthermore, CCD increases the abundance of *L. murinus* in the intestinal tract. The animal experiments confirm that *L. murinus* ameliorates fever in mice.

**Conclusion:**

CCD exhibits remarkable antioxidant and anti-inflammatory properties for effectively treating wind-cold-type common cold. Furthermore, its regulatory effects on *L. murinus* represent a novel mechanism for product development.

## Graphical Abstract

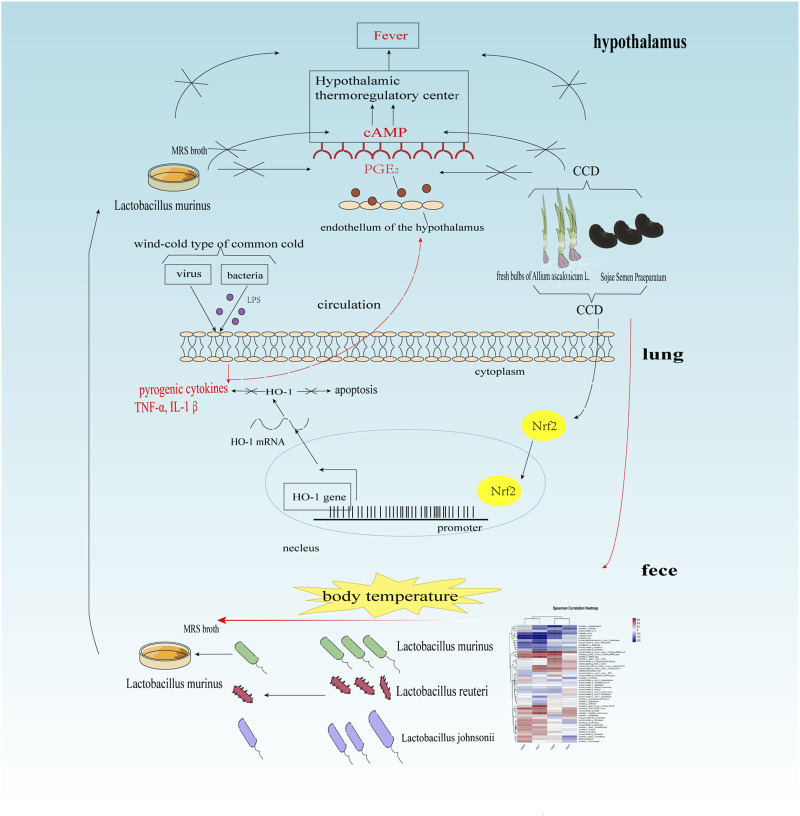

## 1 Introduction

The Cong-Chi decoction (CCD) that contains *Allium ascalonicum* L. (shallot) bulbs and Sojae Semen Praeparatum (SSP) can be traced back to "*Zhouhou Beiji Fang*"—a distinguished compendium known for its comprehensive cataloging of classic medicinal formulations (Huang et al., 2019; Adeyemo et al., 2023). Such botanical drugs have been revered as traditional medicinal foods in China and have been utilized for millennia to address wind-cold, a type of common cold. Current therapeutic strategies for amelioration of the common cold primarily entail administration of non-steroidal anti-inflammatory drugs (NSAIDs), such as acetaminophen. However, extended or unsuitable applications of such pharmacological compounds can cause numerous deleterious effects, including urticaria, liver dysfunction, nausea, and vomiting (Mccrae et al., 2018; Tadokoro-Cuccaro et al., 2022). In contrast to many over-the-counter pharmaceuticals, dietary interventions for treating wind-cold exhibit minimal to no significant adverse effects or reactions (Hai-Long et al., 2015). The principle of "food as medicine” highlights CCD as a therapeutic supplement and promising alternative treatment for fever and inflammation. However, extant studies have primarily focused on shallot bulbs and SSP separately, so the potential synergistic interactions between them in the therapeutic context of wind-cold have been insufficiently examined.

Contemporary medical knowledge highlights the intricate links between phytochemical constituents and therapeutic efficacy. Specifically, shallot bulbs are used to craft functional foods with augmented therapeutic efficacies, which are attributed to the presence of flavor precursors within their cellular matrix (Raeisi et al., 2016; Golubkina et al., 2019; Wenli et al., 2019). The flavor precursor substances within the cellular matrix of shallots, such as S-alk(en)yl cysteine sulfoxide compounds, catalyze enzymes such as alliinase and lachrymatory factor synthase; they also release organic sulfides in conjunction with other bioactive compounds like flavonols and phenols (Malik et al., 2021), thereby imbuing shallot bulbs with distinct antioxidant and anti-inflammatory properties. The regulation of the Nrf2/HO-1 antioxidant signaling pathway by SSP, which diminishes oxidative damage to vascular endothelial cells, is facilitated by the presence of isoflavones such as daidzein and glycitein (Jinzhou et al., 2021). These compounds can activate the upstream Nrf2 pathway and enhance the expression of downstream heme oxygenase-1 (HO-1), thereby mitigating oxidative stress. Nrf2 has been shown to antagonize inflammation, inhibit the expressions of inflammatory mediators, and partially mitigate the inflammatory responses mediated by neutrophils, macrophages, and lymphocytes (Lym) through its downstream protein HO-1 and its metabolites (Gulla et al., 2014; Sivandzade et al., 2019). Specifically, empirical evidence has demonstrated that activation of HO-1 significantly attenuates replication of the influenza virus and ameliorates lipopolysaccharide (LPS)-induced pulmonary damage in rodent models, thereby highlighting the indispensable role of HO-1 as a negative modulator of both inflammatory processes and oxidative stress (Cao et al., 2016; Yin et al., 2017). Consequently, the therapeutic approach of CCD for the wind-cold-type common cold necessitates the cooperative antioxidant and anti-inflammatory actions of shallot bulbs and SSP.

The common cold is an infection of the upper respiratory tract caused by viruses or bacteria. Although self-limiting, it is a highly prevalent condition where adults often experience the common cold two to three times a year, while children under the age of two are susceptible to the infection nearly six times a year (Hai-Long et al., 2015). According to the principles of traditional Chinese medicine (TCM), the common cold is an external ailment that can be categorized into three types: wind-cold, wind-heat, and summer-heat dampness (Jiao et al., 2013). In daily life, the wind-cold-type common cold is a fairly common occurrence, especially during spring and winter. Liu et al. (2021) replicated the symptoms of wind-cold using BALb/c mice by including upregulation of the pro-inflammatory cytokines in the lungs, such as TNF-α, IL-1β, and IL-6 (Jia et al., 2021; Liu et al., 2021), and reported that exposure to cold conditions can induce apoptosis of the lung and spleen cells. Wan et al. (2022) found that rats with wind-cold exhibited some characteristic features such as elevated body temperature, reduced white blood cell (WBC) count, and reduced Lym ratio. Fever is a clinical symptom associated with progressive changes in viral or pathogenic infections; it occurs when a pathogen acts as an external pyrogen to induce release of excessive pyrogenic cytokines in the host immune cells. These cytokines, such as the IL-1β, activate the thermoregulatory center in the body via cAMP and PGE2, resulting in fever (Huang et al., 2020). These data imply that mice with wind-cold have higher levels of cAMP and PGE2. However, these two crucial factors have not been investigated in the aforementioned studies. Xi et al. (2022) administered *Pueraria* root, a traditional medicinal food, to rats with wind-cold and correlated the gut microbiota with body temperature via 16S rRNA sequencing; their findings revealed that rats subjected to wind-cold conditions exhibited notably elevated body temperatures accompanied by reduced *Lactobacillus murinus* populations and concomitant increase in *Prevotella*. This signifies the potential antipyretic efficacy of *L. murinus*. SSP intricately modulates the abundance of six quintessential resident bacteria within the human gastrointestinal microbiome, including *Escherichia coli*, *Enterococcus*, *Bifidobacterium*, *Lactobacillus*, *Clostridium perfringens*, and *Bacteroides* (Yang et al., 2022). Furthermore, the byproducts derived from shallots are shown to significantly foster the proliferation of *Lactobacillus* spp. within the avian gastrointestinal environment (Mozin et al., 2015). However, the potential of CCD to modulate the gut microbiota landscape remains unresolved and merits further exploration. Additionally, evidence suggests that *Lactobacillus* can upregulate the Nrf2/HO-1 signaling pathway and protect against colitis (El-Baz et al., 2020). However, the intricate interplay between the gut microbiota, Nrf2/HO-1 pathway, and inflammatory processes within the wind-cold model remains unclear.

This study comprehensively assesses the therapeutic effects of CCD on wind-cold in mice and elucidates the mechanisms underlying the role of CCD in improving wind-cold symptoms. These mechanisms include modulation of the Nrf2/HO-1 pathway and its impact on the gut microbiota, particularly on the abundance of *L. murinus*.

## 2 Materials and methods

### 2.1 Plant materials and reagents

Fresh shallot bulbs were purchased from Guyu Market, Baiyun District, Guangzhou, Guangdong Province. SSP was prepared and tested as per the procedures of the Chinese Pharmacopoeia at the Heilongjiang Academy of Traditional Chinese Medicine (Heilongjiang, China). The materials were accurately identified by Professor Chao Zhi (School of Traditional Chinese Medicine, Southern Medical University, Guangzhou). Based on converted doses, the experimental mice in this study were categorized into three groups as follows: low dose, 4.1 g/kg (L CCD); medium dose, 8.2 g/kg (M CCD); high dose, 16.48 g/kg (H CCD). SSP (48 g) was placed in 500 mL of water for 1 h and boiled for 20 min. Fresh bulbs of *A. ascalonicum* L (60 g) were then added while boiling, and the mixture was filtered through a double-layered gauze before being concentrated to a final volume of 66 mL. *Fenghan ganmao* granules that are extensively utilized in the treatment of wind-cold were purchased from Haiwang Xingchen Pharmacy in Baiyun District, Guangzhou. These granules are frequently employed in animal studies as the positive control group (Liu et al., 2021; Wan et al., 2022). In addition, an Annexin V-FITC/PI kit (#556547, Becton Dickinson and Company) for determining cellular apoptosis as well as PGE2 (#202301) (#202308-M012), cAMP (#202301) (#202308-M018), IL-6 (#202306-M12), TNF-α (#202306-M22), and IL-1β (#202306-M16) enzyme-linked immunoassay (ELISA) kits were purchased from Jiangsu Mmbio Co., Ltd. Antibodies against HO-1 (#AF5393) and Nrf2 (#AF0639) were lastly purchased from Affinity Biosciences (United States).

### 2.2 Animals

The study mice were housed in a controlled environment with a pressure ventilation system at a relative humidity of 50% ± 5% and an ambient temperature of 25°C ± 2 °C. The mice were exposed to a 12 h/12 h light/dark cycle and provided free access to water and a regular diet. The animals were acclimated to the environment for roughly 3 days prior to experimentation. The animal study protocols were approved by the Animal Ethics Committees of the Institute of Biological and Medical Engineering of Guangdong Academy of Sciences. All animal studies were carried out in compliance with NIH regulations (ethics approval number: K2022-01-032-077).

### 2.3 Study design

**FIGURE 1:**
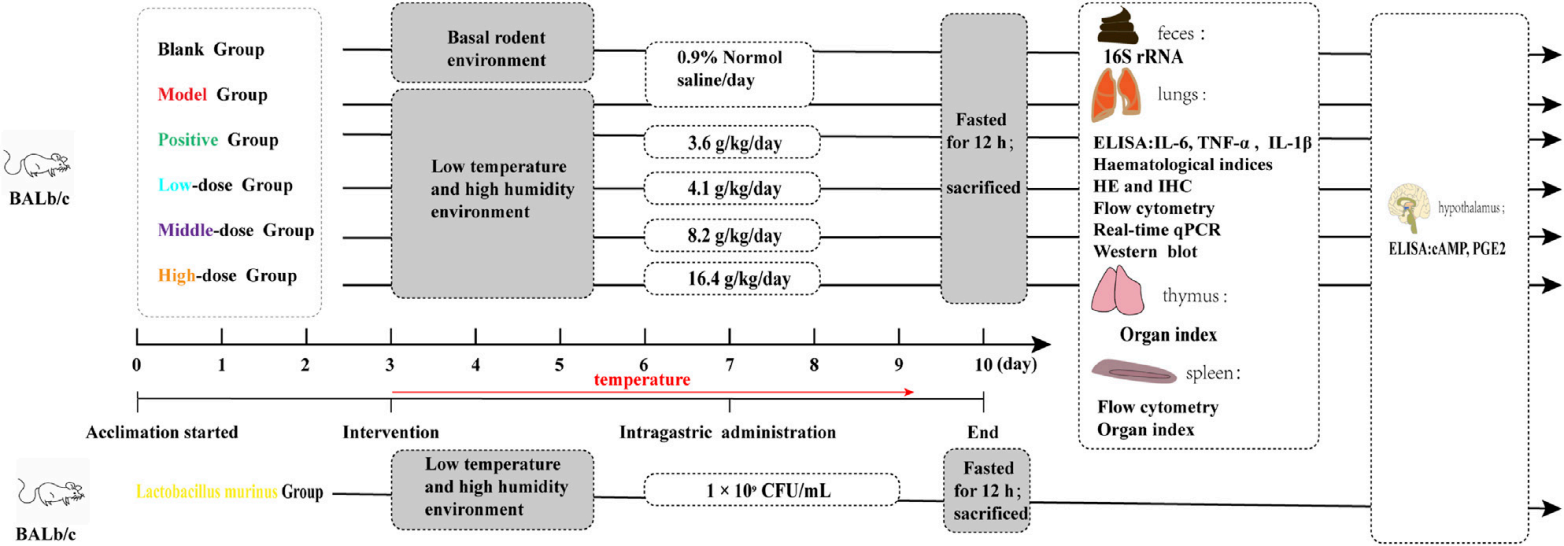
Experimental groups and associated therapies.

The experimental groups and corresponding treatments are shown in Figure 1. In this study, male BALb/c mice were induced to develop wind-cold. The mice were then administered varying dosages of CCD or *F. ganmao* granules after a period of acclimation. The study design consisted of six groups: blank, model, positive (3.6 g/kg/day), high dose (16.4 g/kg/day), medium dose (8.2 g/kg/day), and low dose (4.1 g/kg/day). These mice were caged in small animal incubators (TPC 460, ALISN Shanghai Co., Ltd.) separate from the blank control and maintained at a cool temperature (2°C–5°C) under a 12 h/12 h light/dark cycle and humidity of 60% ± 5%. Then, CCD or *F. ganmao* granules were administered to the mice from day 4 and continued for three consecutive days, and their body temperatures were assessed each day. The mice were then fasted for 12 h and euthanized to obtain blood, lung, spleen, thymus, hypothalamus, and feces samples, which were stored at −80°C until examination.

### 2.4 Temperature indicators

After the first 3 days of acclimation, the body temperatures of the mice were recorded daily.

### 2.5 Hematological indicators

The WBCs and Lym were measured using a BC-5000 vet automatic blood cell analyzer (Shenzhen Mindray Bio-Medical Electronics Co., Ltd., Nanshan, Shenzhen, China).

### 2.6 Determination of organ indices

The collected spleen, thymus, and other organs were dissected immediately, and the connective tissue and fat were removed rapidly. The organs were rinsed with phosphate-buffered saline (PBS) and dried using clean dust-free absorbent paper. The weight of each organ was measured using an analytical scale, and the organ index was calculated as the ratio of the organ wet weight to total body weight.

### 2.7 Hematoxylin and eosin (H&E) staining

The collected lung tissues were fixed in 4% paraformaldehyde. After paraffin embedding, 4-μm-thick tissue sections were cut and dewaxed sequentially, stained with hematoxylin, differentiated with hydrochloric acid alcohol, and then stained with eosin.

### 2.8 Detection of cellular apoptosis in the lungs and spleen by flow cytometry

Single-cell suspensions were prepared from the collected tissue samples using the mesh rubbing method. The red blood cells were lysed, and the cell concentration was adjusted to 10^5^ cells/mL. Then, the Annexin V-FITC/PI kit was used to measure apoptosis. The cells were mixed with a buffer, Annexin V-FITC, and propidium iodide according to manufacturer instructions, followed by incubation on ice in the dark for 15 min. Flow cytometry was performed using BD FACS Canto II (Becton Dickinson and Company) and NOVOEXPRESS.

### 2.9 Enzyme-linked immunosorbent assay

The collected hypothalamic and lung tissues were homogenized in lysis buffer. Then, the levels of cAMP, PGE2, IL-6, TNF-α, and IL-1β were measured by ELISA according to manufacturer instructions.

### 2.10 Immunohistochemical analyses

The tissue sections prepared earlier (as noted in Section 2.7) were immersed in a solution containing HO-1 and Nrf2 antibodies (1:1500 dilution, 100 μL), and a universal detection kit was used as the secondary antibody. Tap water was used to halt color development. The nuclei in the slides were counterstained, followed by dehydration and air-drying using the gradient alcohol method. Finally, the slides were mounted to obtain images using a MoticEasyScan scanner (Motic Company, China). The sections were assessed using Image-Pro Plus 6.0 to quantify the positive cell counts at a magnification of ×50.

### 2.11 Quantitative real-time polymerase chain reaction assay

The mRNA levels of different genes were analyzed by qRT-PCR using a SYBR Green Kit (Toyobo, Japan). The total RNA was extracted from the lung tissues using TRIzol (Invitrogen, Carlsbad, CA, United States). The primer sequences used for RT-qPCR are listed in Table 1. The expression levels of all mRNA genes were normalized to the endogenous β-actin expression level and calculated using the 2^-ΔΔCT^ method.

**TABLE 1:** Primer sequences.

Gene	Forward primer (5'->3′)	Reverse primer (3'->5′)
Nrf2	TAG​ATG​ACC​ATG​AGT​CGC​TTG​C	GCC​AAA​CTT​GCT​CCA​TGT​CC
HO-1	GAT​AGA​GCG​CAA​CAA​GCA​GAA	CAG​TGA​GGC​CCA​TAC​CAG​AAG
β-actin	CTCTCCCTCACGCCATC	ACGCACGATTTCCCTCTC

### 2.12 Western blot

A 20-mg sample of lung tissue was processed at a low temperature using a KZ-II high-speed tissue grinder (Wuhan Sevier Biotechnology Co., Ltd.). Then, total protein extraction was performed using RIPA buffer, and the protein concentration was determined using a BCA kit. Following normalization of the protein concentration, the proteins were denatured at 95°C. SDS-PAGE gels of various concentrations were prepared based on the protein molecular weights. Electrophoresis was used to separate the Nrf2 and HO-1 proteins, which were subsequently transferred onto a polyvinylidene fluoride (PVDF) membrane and incubated at room temperature. The separated bands were blocked, washed, and incubated overnight with Nrf2, HO-1, and β-actin antibodies at 4°C. After washing, the membrane was exposed to horseradish peroxidase (HRP)-conjugated secondary anti-rabbit IgG (1:4000 dilution) for 1 h at room temperature, followed by further washing. Finally, the signals were visualized using a gel imager (BIO-RAD, United States) and analyzed using ImageJ 1.52 software.

### 2.13 16S rRNA gene sequencing

The 16S rRNA gene sequencing in this study was performed with the assistance of Majorbio Biotechnology Co., Ltd. (Shanghai, China).

### 2.14 *Lactobacillus murinus* treatment

The cloud platform designed by Majorbio Biotechnology Co., Ltd. was used to select and correlate *L. murinus* levels with the body temperatures of the mice. The taxonomic classification of *L. murinus* was established via complete genome sequencing. The strain was incubated at 37°C for 24 h until the late logarithmic growth phase was reached. The colonies were counted to ascertain the concentration of viable bacteria, which was determined to be 10^9^ CFU/mL (Li et al., 2023). Following the removal of the MRS broth, the *L. murinus* treatment group was administered 10^9^ CFU/mL of *L. murinus* in 0.1 mL of diluted physiological saline orally for four consecutive days. The experimental groups and their respective treatments are depicted in Figure 1.

### 2.15 Statistical analysis

The data are presented as the mean ± standard error of the mean (SEM). SPSS 2.0 (IBM, Armonk, New York, United States) and GraphPad Prism (version 8.0) were used for the statistical analyses. One-way ANOVA was used to compare the parametric data across groups after verification of the homogeneity of variance, and pairwise comparisons were performed using the least significant difference method. The Kruskal–Wallis test was used to evaluate the statistically significant differences between the groups for the non-parametric data. The Spearman correlation coefficient test was applied to explore the relationships between the gut microbiota and body temperatures. The significance level was set at *p* < 0.05.

## 3 Results

### 3.1 Effects of CCD on temperature and hypothalamic cAMP and PGE2 in BALb/c mice

**FIGURE 2:**
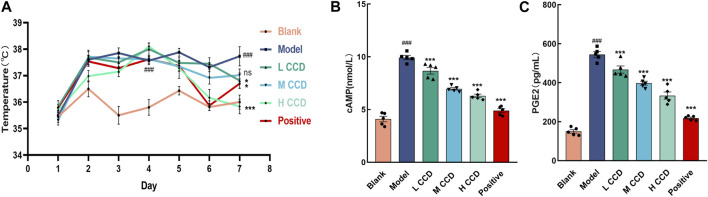
Effects of CCD on body temperature and hypothalamic cAMP and PGE2: **(A)** temporal profile illustrating changes in the body temperatures of mice in each group over a 7-day period (n = 6); **(B, C)** CCD administration resulted in significant inhibition of cAMP and PGE2 secretions in the hypothalamic tissues of the model mice (*n* = 5). All data are expressed as the mean ± standard error of the mean. ^##^ *p* < 0.01 and ^###^ *p* < 0.001 compared with the blank group. *p < 0.05, **p < 0.01, and ***p < 0.001 compared with the model group.

The body temperatures of the mice were monitored over a span of seven consecutive days (Figure 2A). The body temperatures exhibited significant increases in both the model and dosing groups, with noticeable differences compared to the blank group before treatment (*p* < 0.001). In the final temperature measurements, the model group exhibited a significant increase over the blank group (*p* < 0.001). When compared with the model group, the body temperatures of the groups with high and low levels of CCD and those of the positive group decreased significantly (*p* < 0.001, *p* < 0.05, and *p* < 0.05, respectively). Furthermore, the levels of cAMP (Figure 2B) and PGE2 (Figure 2C) were significantly higher in the model group than the blank group (*p* < 0.001 and *p* < 0.001, respectively). However, treatment with CCD at three different doses significantly reduced the levels of cAMP and PGE2 in the hypothalamus compared with those of the model group (*p* < 0.001, *p* < 0.001, and *p* < 0.001; *p* < 0.001, *p* < 0.001, and *p* < 0.001, respectively). Furthermore, compared to the model group, the positive group exhibited significant reductions in both cAMP and PGE2 (*p* < 0.001 and *p* < 0.001, respectively). These results indicate that the CCD treatment was effective at reducing pyrexia.

### 3.2 Effects of CCD on inflammatory factors in the lung tissues of BALb/c mice

**FIGURE 3:**
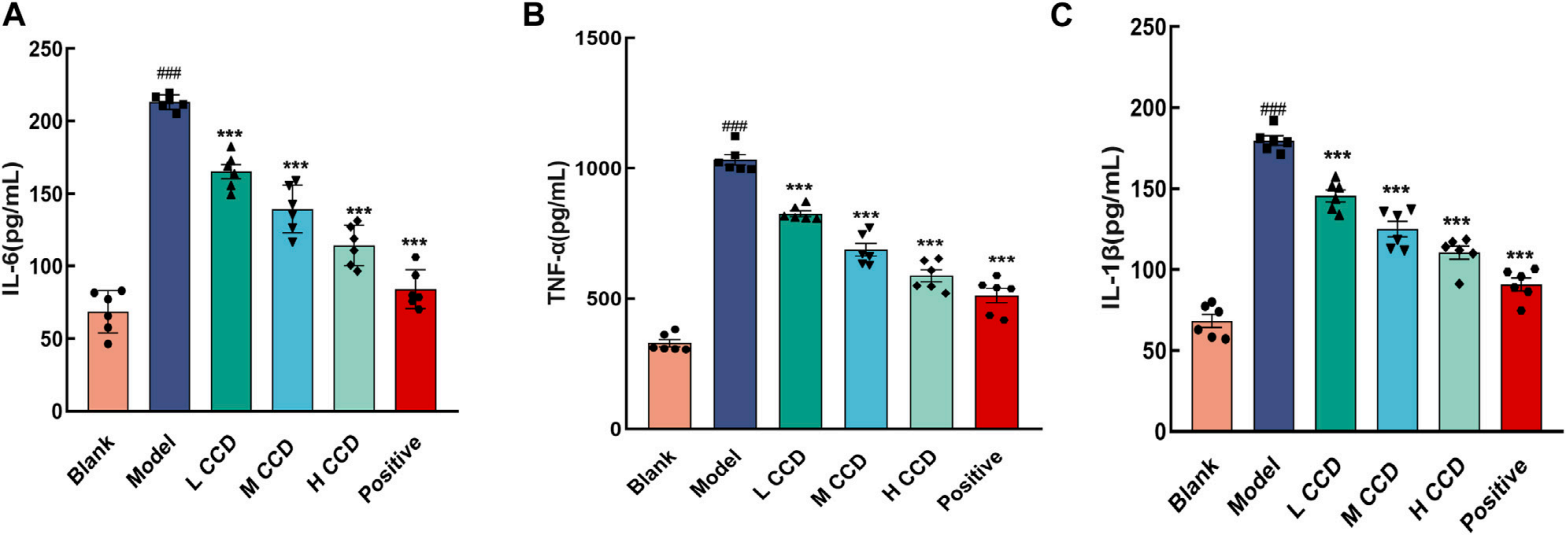
Effects of CCD on inflammatory factors in the lung tissues of BALb/c mice. CCD administration resulted in the suppression of TNF-α, IL-6, and IL-1β secretions in the lung tissues of model mice (*n* = 6). Secretions of **(A)** IL-6, **(B)** TNF-α, and **(C)** IL-1β. All data are expressed as the mean ± standard error of the mean. ^###^ *p* < 0.001 compared with the blank group. ***p < 0.001 compared with the model group.

In the model group, the levels of IL-6 (Figure 3A), TNF-α (Figure 3B), and IL-1β (Figure 3C) significantly exceeded the levels of the blank group (*p* < 0.001, *p* < 0.001, and *p* < 0.001), indicating abnormal inflammatory responses in the experimental mice. Following 3 days of CCD treatment at various doses, the inflammation levels exhibited clear dose–response relationships. Compared with the model group, the IL-1β, TNF-α, and IL-6 levels decreased significantly in the groups treated with low, medium, and high doses of CCD as well as in the positive group (*p* < 0.001, *p* < 0.001, *p* < 0.001, and *p* < 0.001, respectively). These findings demonstrate that CCD treatment effectively improved inflammatory levels in the model group.

### 3.3 Effects of CCD on hematological indices

**FIGURE 4:**
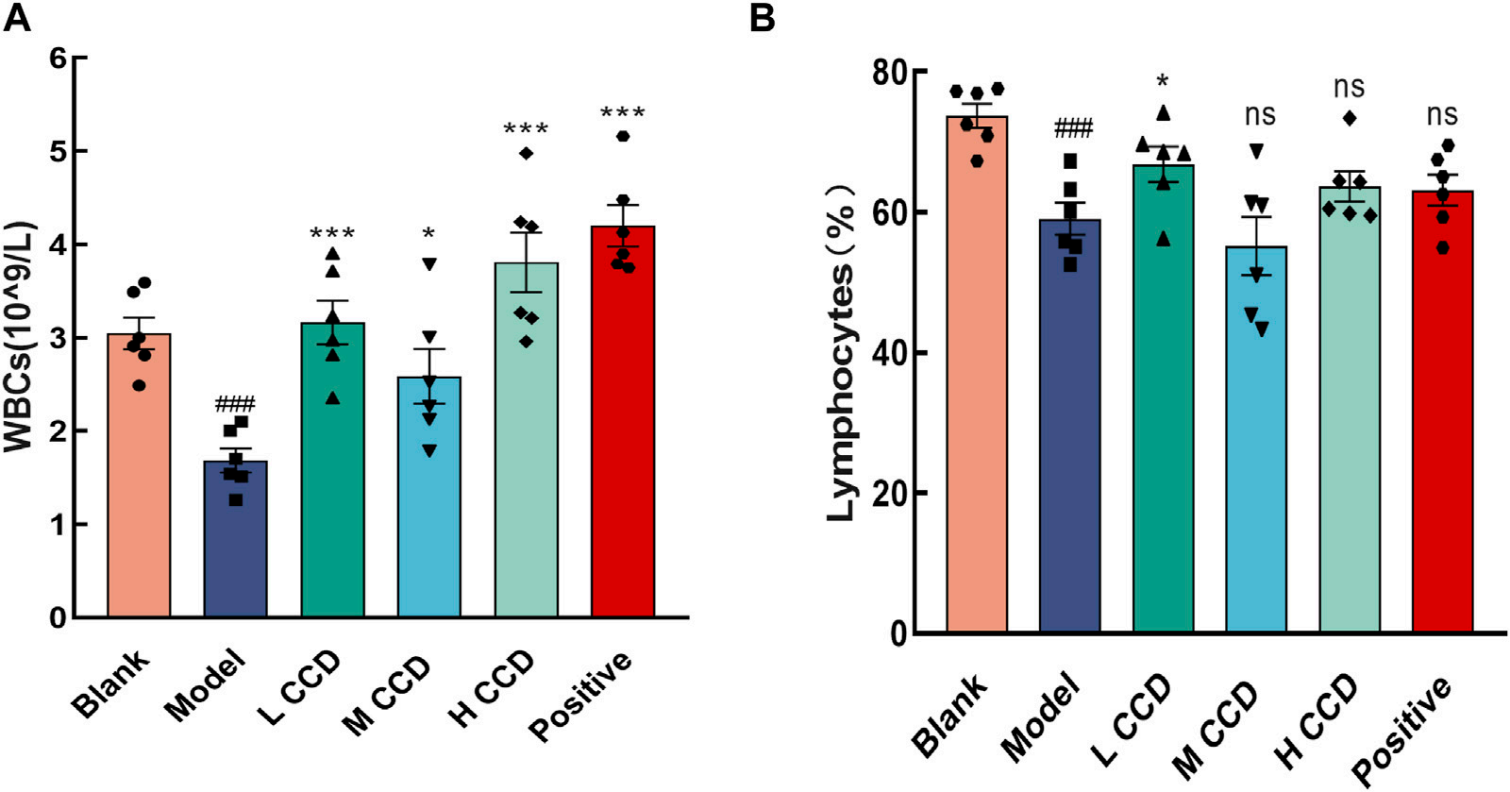
Effects of CCD on hematological indices. CCD increased the white blood cell count and lymphocyte ratio in the model mice (*n* = 6). Changes in the **(A)** white blood cell count and **(B)** lymphocyte ratio. All data are expressed as the mean ± standard error of the mean. ^###^ *p* < 0.001 compared with the blank group. *p < 0.05, and ***p < 0.001 compared with model group.

The WBC count (Figure 4A) and Lym ratio (Figure 4B) were significantly lower in the model group than the blank group (*p* < 0.001 and *p* < 0.001, respectively). All treated groups showed significant increases in the WBC counts (*p* < 0.001, *p* < 0.05, *p* < 0.001, and *p* < 0.001, respectively), while the low CCD group exhibited a significant increase in the Lym ratio (*p* < 0.05) than the model group.

### 3.4 Histopathological examination

**FIGURE 5:**
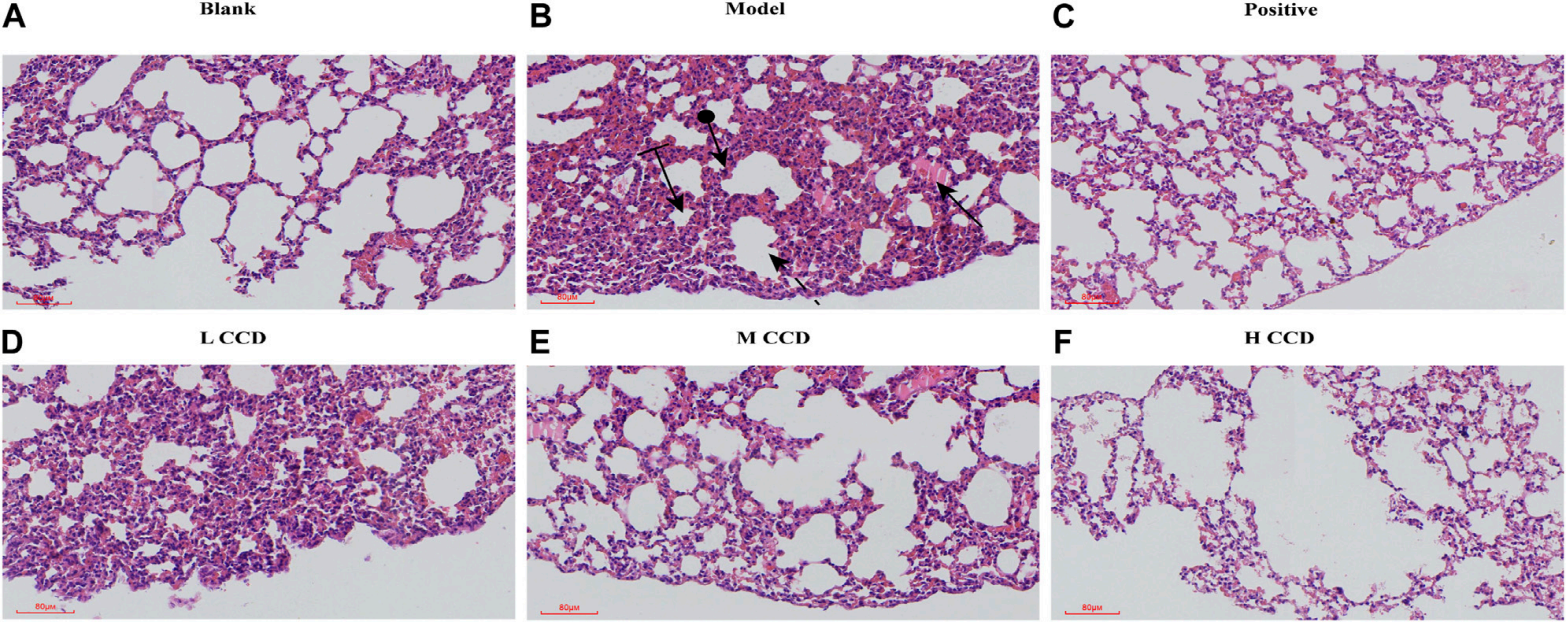
Effects of CCD on lung histopathology in mice in the **(A)** blank group (H&E, × 80) and **(B)** model group (H&E, × 80), demonstrating neutrophil infiltration (round arrow), hemorrhage (flat arrow), compensatory vacuole (striped arrow), and interstitial proliferation (dashed arrow). Corresponding lung histopathologies in the **(C)** positive group (H&E, × 80), **(D)** low-dose group (H&E, × 80), **(E)** medium-dose group (H&E, × 80), and **(F)** high-dose group (H&E, × 80). The histological sections were stained with hematoxylin and eosin (H&E) to visualize tissue morphology at ×80 magnification.

The results of the histological examination of the lung tissue samples are shown in Figure 5. The model group showed pulmonary interstitial hyperplasia, thickening of the vascular walls, local hemorrhage, and infiltration of neutrophils. Additionally, modern compensated vacuoles were observed occasionally. However, after treatment with CCD at different doses, these pathological features were blocked.

### 3.5 Effects of CCD on organ indices

The results for the organ indices are shown in Table 2. Compared to the blank group, the model group exhibited significant decreases in the spleen and thymus indices (*p* < 0.001 and *p* < 0.05, respectively). Compared with the model group, the spleen indices of the medium-dose CCD, positive, and high-dose CCD groups were significantly higher (*p* < 0.05, *p* < 0.001, and *p* < 0.001, respectively), while the thymus index was significantly higher in the high-dose group (*p* < 0.05).

**TABLE 2:** Effects of different treatments on the mouse organ indexes.

Group name	Spleen index	Thymus index
Blank group	0.3774 ± 0.0345	0.1979 ± 0.0191
Model group	0.3009 ± 0.0422^b^	0.1667 ± 0.0238^a^
Low-dose CCD	0.3164 ± 0.0234	0.1739 ± 0.0243
Middle-dose CCD	0.3226 ± 0.0318*	0.1882 ± 0.0263
High-dose CCD	0.3616 ± 0.0493***	0.1959 ± 0.0635*
Positive group	0.3590 ± 0.0423***	0.1954 ± 0.0424*

Notes: Values are presented as mean ± SEM; *n* = 6.

^a^ *p <* 0.05.

^b^ *p <* 0.001, *p < 0.05, and ***p < 0.001 compared with the model group.

### 3.6 Effects of CCD on cellular apoptosis of the lungs and spleen

**FIGURE 6:**
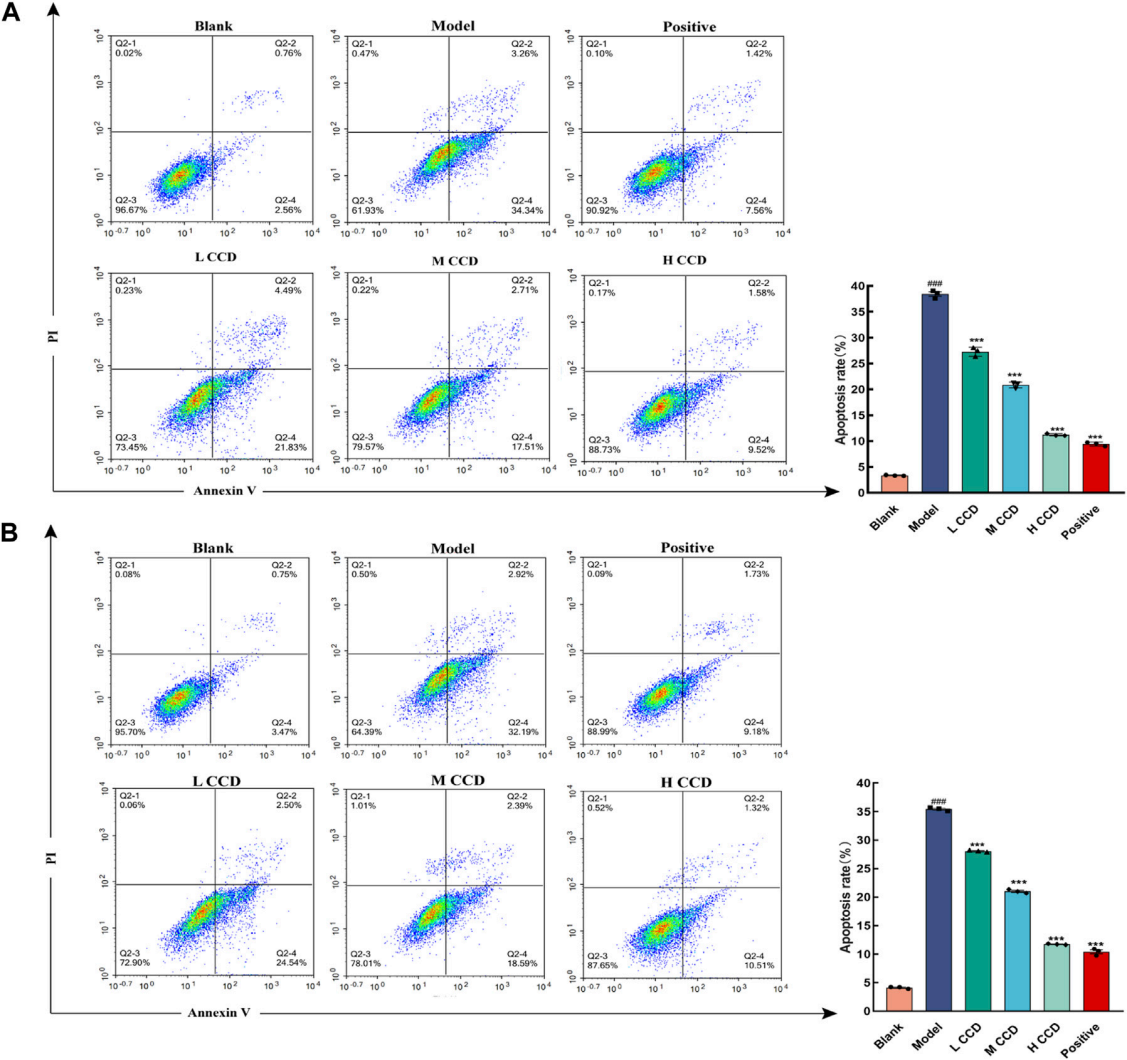
Effects of CCD showing inhibition of cellular apoptosis of the lungs and spleen (*n* = 3). Flow cytometry scatterplots of **(A)** mouse lung cells and **(B)** spleen cells. All data are expressed as the mean ± standard error of the mean. ^###^ *p* < 0.001 compared with the blank group. ***p < 0.001 compared with the model group.

The results of cell apoptosis are shown in Figure 6. Wind-cold significantly increased the degrees of apoptosis in the primary lung cells and primary splenocytes when compared with the blank group of mice (*p* < 0.001 and *p* < 0.001, respectively). However, all treated groups exhibited significant reductions in cellular apoptosis of the lungs (*p* < 0.001, *p* < 0.001, *p* < 0.001, and *p* < 0.001) and spleen (*p* < 0.001, *p* < 0.001, *p* < 0.001, and *p* < 0.001). The positive group showed the greatest reduction, followed by the high- and medium-dose groups.

### 3.7 CCD treatment increases expressions of Nrf2 and HO-1 in the lung tissues of BALb/c mice

**FIGURE 7:**
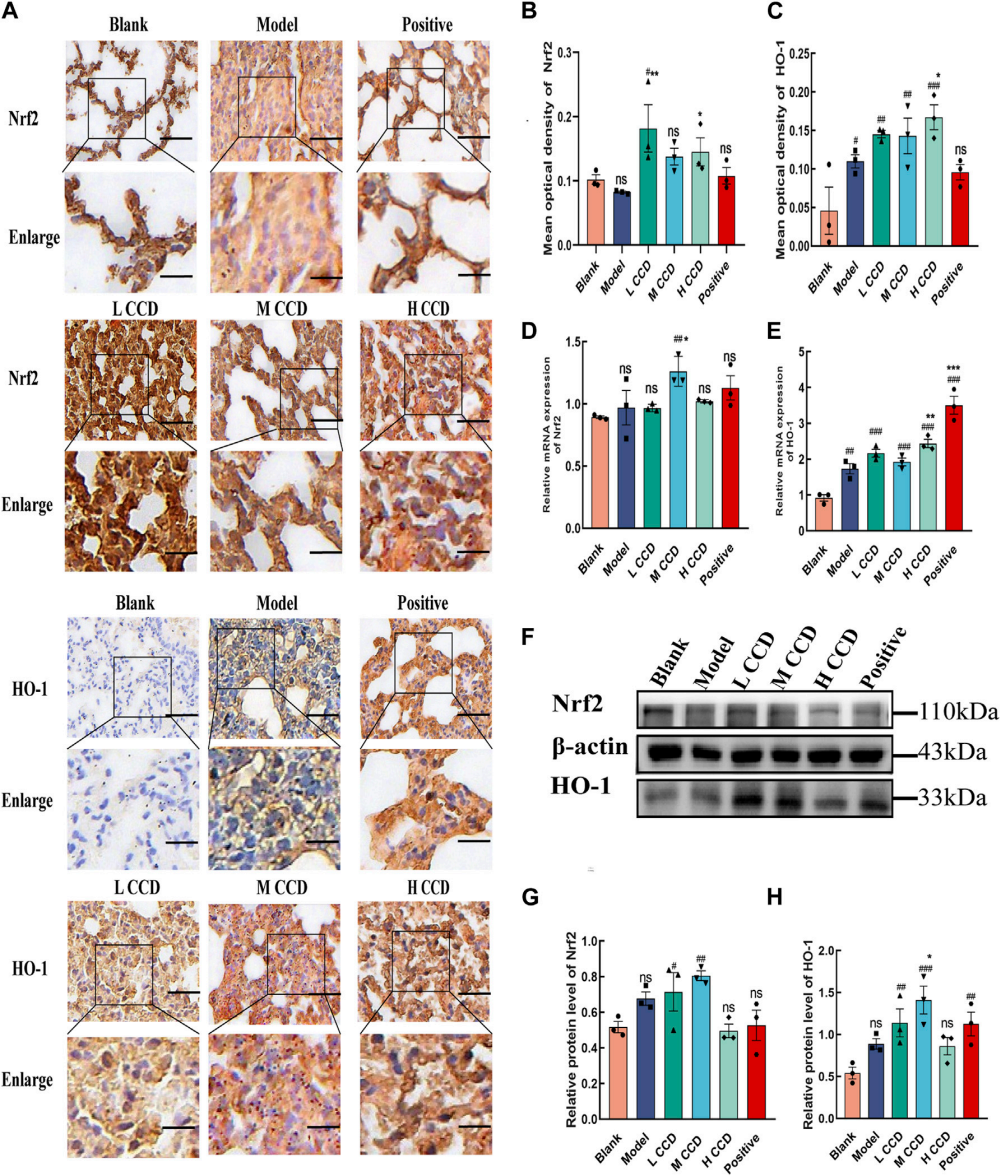
CCD treatment increased the expressions of Nrf2 and HO-1 in the lung tissues of BALb/c mice (*n* = 3): **(A–C) i**mmunohistochemistry, **(D–E)** quantitative real-time PCR, and **(F–H)** Western blot results showing the effects of CCD on the expressions of Nrf2 and HO-1 in the lung tissues of BALb/c mice. Scale bar: 50 µm. All data are expressed as the mean ± standard error of the mean. ^#^ *p* < 0.05, ^##^ *p* < 0.01, and ^###^ *p* < 0.001 compared with blank group *p < 0.05, **p < 0.01, and ***p < 0.001 compared with the model group.

To investigate whether CCD improved wind-cold by altering the redox status, the Nrf2/HO-1 signaling pathway was analyzed. Immunohistochemical staining of the lung tissues obtained from BALb/c mice revealed the effects of CCD on the expressions of Nrf2 and HO-1, as shown in Figure 7A (scale bar: 50 µm). The results showed that for the medium-dose group, the mean optical density of Nrf2 (Figure 7B) surpassed those of the blank and model groups (*p* < 0.05, *p* < 0.01). Additionally, for the high-dose group, the mean optical density of Nrf2 (Figure 7B) exceeded that of the model group (*p* < 0.05). Moreover, the mean optical densities of HO-1 in both the CCD and model groups (Figure 7C) were higher than that in the blank group (*p* < 0.05, *p* < 0.01, *p* < 0.01, and *p* < 0.001). There was also an increasing trend in the high-dose group compared to the model group (*p* < 0.05). The medium-dose group exhibited significantly increased relative mRNA expression of Nrf2 (Figure 7D) (blank group vs. medium-dose group, *p* < 0.001) (model group vs. medium-dose group, *p* < 0.01). Compared to the blank group, both the model and treatment groups exhibited significant upward trends in the relative mRNA expressions of HO-1 (Figure 7E) (*p* < 0.01, *p* < 0.001, *p* < 0.001, *p* < 0.001, and *p* < 0.001). Additionally, when compared to the model group, the high-dose and positive drug groups also showed increasing trends (*p* < 0.01 and *p* < 0.001, respectively). The Western blot (Figures 7F–H) analysis revealed that exposure to CCD increased the expression of Nrf2 in lung tissue (blank group vs. low-dose group, *p* < 0.05) (blank group vs. medium-dose group, *p* < 0.01). Compared to the blank group, both the low- and medium-dose groups exhibited significant upward trends in the expressions of HO-1 (*p* < 0.01 and *p* < 0.001, respectively). Furthermore, compared to the model group, the mid-dose group showed an increasing trend (*p* < 0.05). Hence, the CCD treatment was concluded to activate the Nrf2/HO-1 signaling pathway and prevent redox imbalance in mice with wind-cold.

### 3.8 Antipyretic effects of CCD are positively correlated with *L. murinus* abundance

**FIGURE 8:**
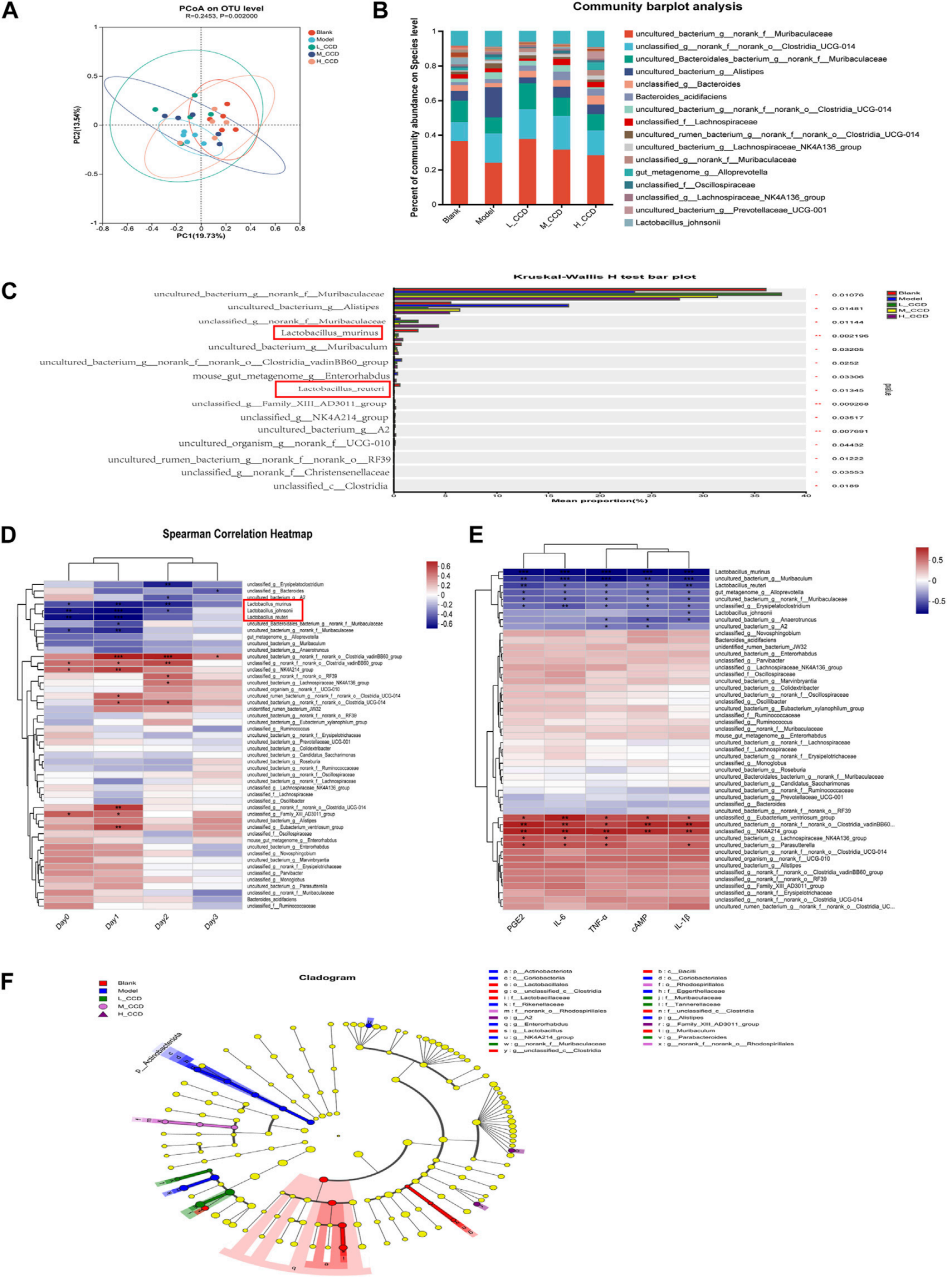
Effects of CCD on the gut microbiota (*n* = 5). **(A)** Unweighted UniFrac principal coordinates analysis (PCoA) based on OTUs. **(B)** Percentage of community abundance at the species level. **(C)** Top 15 abundant species showing differences in abundance in the blank, model, high-dose, medium-dose, and low-dose groups. **(D)** Heatmap showing hierarchical clustering between the fecal bacterial abundance and body temperatures of mice using the Spearman correlation coefficient. **(E)** Heatmap showing hierarchical clustering between the fecal bacterial abundance and selected cytokine levels using Spearman correlation coefficient. **(F)** LEfSe was employed to analyze the dominant bacteria at various taxonomic levels (from order to species) among the five groups. The Kruskal–Wallis test was used to evaluate statistical significance between the groups for non-parametric data. The Spearman correlation coefficient test was applied to explore the relationships between the gut microbiota and body temperatures. The significance level was set at *p < 0.05, **p < 0.01, and ***p < 0.001.

The beta-diversity-based principal coordinate analysis (PCoA) is presented in Figure 8A. The first principal coordinate (PC1) accounted for 13.54% of the intergroup variation, while the second principal coordinate (PC2) contributed to 19.73%. The analysis of similarities (ANOSIM), which is based on the evaluation of intergroup similarities, yielded a result of R = 0.2453 and *p* = 0.002. The top 20 taxa were detected at the species level, as shown in Figure 8B. Differential abundance analysis was performed to compare the abundances among the groups. Compared with the blank group, the model group exhibited significant decreases in the relative abundances of *L. murinus* (*p* < 0.01), *L. reuteri* (*p* < 0.5), and uncultured_bacterium_g__norank_f__*Muribaculaceae* (*p* < 0.5). Conversely, the relative abundances of Uncultured_bacterium_g__*Alistipes* (*p* < 0.5) and mouse_gut__metagenome_g__*Enterorhabdus* (*p* < 0.5) increased (Figure 8C).

The heatmap illustrates the hierarchical clustering between the fecal bacterial abundances and body temperatures of mice using the Spearman correlation coefficient (Figure 8D). A total of 18 species were significantly correlated with the body temperatures, with 10 species showing positive correlations and eight species showing negative correlations. The species positively correlated with body temperature were as follows: uncultured_bacterium_g__norank_f__norank_o__*Clostridia_vadin*BB60_group, unclassified_g__norank_f__norank_o__*Clostridia_vadin*BB60_group, unclassified_g__NK4A214_group, unclassified_g__norank_f__norank_o__RF39, uncultured_bacterium_g__*Lachnospiraceae*_NK4A136_group, uncultured_rumen_bacterium_g__norank_f__norank_o__*Clostridia*_UCG-014, uncultured_bacterium_g__norank_f__norank_o__*Clostridia*_UCG-014, unclassified_g__norank_f__norank_o__*Clostridia*_UCG-014, unclassified_g__Family_XIII_AD3011_group, and unclassified_g__*Eubacterium_ventriosum*_group. The species negatively correlated with body temperature were as follows: unclassified_g__*Erysipelatoclostridium*, unclassified_g__*Bacteroides*, uncultured_bacterium_g__A2, *L*. *murinus*, *L*. *johnsonii*, *L*. *reuteri*, uncultured_*Bacteroidales*_bacterium_g__norank_f__*Muribaculaceae*, and uncultured_bacterium_g__norank_f__*Muribaculaceae*. The anti-inflammatory and antipyretic effects of *L*. *murinus* were further validated, as shown in Figure 8E. In summary, the antipyretic effect of CCD was positively correlated with the abundance of *L*. *murinus*.

Linear discriminant analysis effect size (LEfSe) (Figure 8F) was used to depict the dominant bacteria at various taxonomic levels (from order to species) among the five groups. Only taxa with a linear discriminant analysis (LDA) score greater than two are displayed. The circles represent the phylogenetic levels, with the diameter and color of each circle representing its abundance and group, respectively. As shown in the figure, Lactobacillales and *Bacilli* were predominant in the blank group, *Actinobacteriota* was predominant in the model group, while Muribaculaceae and Tannerellaceae were abundant in the low-dose CCD group. The medium-dose CCD group was dominated by Rhodospirillales, and the high-dose CCD group exhibited mostly *Family_XIII_AD3011_*group.

### 3.9 Oral intake of *L. murinus* alleviates fever in BALb/c mice

**FIGURE 9:**
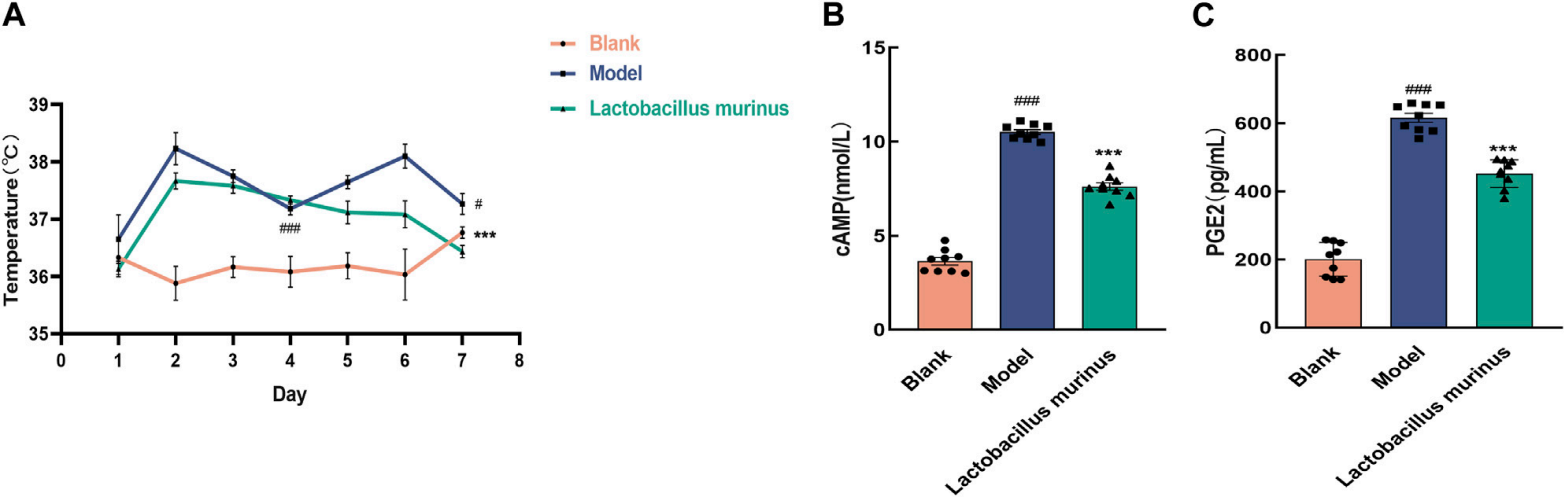
Effects of *L. murinus* on body temperature and hypothalamic cAMP and PGE2 levels. **(A)** Changes in the body temperatures of mice in each group over 7 days (*n* = 6). **(B, C)** *L. murinus* inhibited the secretions of cAMP and PGE2 in the hypothalamic tissues of the model mice (*n* = 9). All data are expressed as the mean ± standard error of the mean. ^#^ *p* < 0.05, ^##^ *p* < 0.01, and ^###^ *p* < 0.001 compared with the blank group. ***p < 0.001 compared with the model group.

The temperatures of the mice were measured for 7 days (Figure 9A). The body temperatures of the mice in both the model and treatment groups increased after commencement of modeling. Before treatment, a significant difference in body temperature was found compared to the blank group (*p* < 0.001). After treatment, the body temperatures decreased (model group vs. *L. murinus*, *p* < 0.001). The results of ELISA are shown in Figures 9B, C. The levels of cAMP and PGE2 were significantly higher in the model mice (model group vs. blank group, *p* < 0.001). Oral intake of *L. murinus* attenuated the elevated levels of cAMP and PGE2, especially in the model mice (model group vs. *L. murinus*, *p* < 0.001).

## 4 Discussion

Currently, functional foods are receiving significant attention, and CCD that is composed of two traditional medicinal foods is the main goal of this investigation. Regarding the development of SSP, the present research group has conducted extensive investigations over the course of nearly 8 years, with the work spanning a wide array of subjects, including fermentation of soy products, selection and identification of probiotic strains, key fermentation technologies, and analysis of fermentation products and their functional properties. Throughout these years, the authors have implemented numerous national scientific research projects as well as achieved a highly stable and mature technology for the preparation of SSP (fermented *Glycine max* (L.) Merr). This has culminated in the formation of a distinct industry. Inspired by the ancient text "*Zhouhou Beiji Fang,*” the present team has achieved innovations by integrating SSP with shallot bulbs to formulate the CCD. The authors have delved deeply into the mechanisms by which CCD ameliorates wind-cold and provided a novel direction for the their product pipeline. Utilizing ultraperformance liquid chromatography tandem mass spectrometry (UPLC-MS/MS), the chemical composition of CCD (S1) was elucidated and its components, including daidzein, genistein, glycitein, and S-methyl-L-cysteine-S-oxide, were characterized via tandem mass spectrometric fragmentation patterns (S2). These components were identified using Compound Discoverer 3.3 software and its database suite, which includes ChemSpider, mzCloud, mzVault, and OTCML. It merits emphasis that these databases are extensively leveraged to elucidate the components used in TCM (Qian et al., 2022; Fan et al., 2024). To investigate the associated pharmacological effects, wind-cold modeling equipment (TPC 460, ALISN Shanghai Co., Ltd.) was used to establish a standardized mouse model for investigating the anti-inflammatory, antioxidant, and antiapoptotic effects of CCD on wind-cold.

In the present study, the authors meticulously measured body temperatures and evaluated the expression levels of the critical temperature-regulating factors cAMP and PGE2 in the hypothalamic region. Most infections occur outside the brain, suggesting that cytokines and bacterial products stimulate hypothalamic neurons (El-Radhi, 1994). The synthesis and release of PGE2 into the brain in response to pyrogenic cytokines is a crucial event at the hypothalamic–endothelial blood–brain barrier (Dinarello et al., 1999). PGE2 alone cannot activate the hypothalamic neurons; instead, it stimulates the release of the neurotransmitter cAMP, ultimately resulting in a fever (Dinarello et al., 1999). An increase or decrease in body temperature is in line with the levels of cAMP and PGE2 in the hypothalamus. Since 2002, hypothalamic cAMP and PGE2 levels have been used to study the antipyretic effects of baicalin (Zhao et al., 2002). In mice with wind-cold, the levels of cAMP and PGE2 in the hypothalamus are significantly higher than in the normal group, indicating increased body temperature due to cold exposure. The treatment with CCD and *L. murinus* led to decreases in the release of cAMP and PGE2 as well as return of body temperature to normal levels. This indicates that both CCD and *L. murinus* have antipyretic (fever-reducing) effects. The common cold is primarily caused by viral or bacterial infections. LPS from the outer membrane of Gram-negative bacteria stimulates the immune system, leading to an inflammatory response in the body along with the release of TNF-α, IL-1β, and IL-6. These inflammatory factors can result in various symptoms, including fever.

Analysis of the pulmonary pathology in the mice showed that the degrees of lung tissue damage and inflammatory cell infiltration were alleviated after CCD administration, indicating that CCD prevented lung injury. In addition, the WBC count and Lym ratio were significantly lower in the model group, consistent with the observations of other published studies (Wan et al., 2022) and indicating an acute inflammatory response. The thymus and spleen indices in the model group were significantly lower than those in the blank group, indicating that CCD enhanced immune function and inhibited inflammatory responses. Jia et al. (2021b) reported that exposure of the body to cold conditions induces lung cell apoptosis. Simultaneously, as the spleen is the largest peripheral immune organ, the body can induce immune responses to external pathogens, resulting in the apoptosis of spleen cells. The present investigation revealed that CCD therapy significantly mitigated cold-induced apoptosis and inflammatory responses, potentially through the regulatory mechanisms of the Nrf2/HO-1 pathway. It has been reported that activation of the Nrf2/HO-1 pathway is associated with reduced oxidative stress as well as decreased inflammation and apoptosis (Yang et al., 2022). The findings of this study indicate that the expression levels of Nrf2 and HO-1 are modestly elevated in the lung tissue of the model group, a trend that is frequently observed in LPS-induced acute lung injury. LPS-induced acute lung injury causes increased expressions of Nrf2 and HO-1; cordycepin and panaxydol have been shown to mitigate LPS-induced inflammation by sustained upregulation of Nrf2 and HO-1 expressions (Qing et al., 2018; Li et al., 2021). Jia et al. (2021a) attributed the seemingly unchanged levels of Nrf2 and HO-1 to intrinsic self-protective mechanisms. The organism coordinates its responses to cold-induced pulmonary injuries through moderate activation of the Nrf2 and HO-1 pathways, although the balance between the synthesis and utilization of Nrf2 and HO-1 alone is insufficient to halt the progression of lung damage. Following CCD administration, enhanced upregulation of Nrf2 and HO-1 was observed, suggesting that CCD may activate the Nrf2/HO-1 pathway, thereby augmenting gene expression and facilitating the synthesis and secretion of the Nrf2/HO-1 proteins. Studies indicate that activation of the Nrf2/HO-1 pathway can effectively attenuate pro-inflammatory mediators (Loboda et al., 2016; Chen et al., 2019). Furthermore, the activation of the Nrf2/HO-1 pathway plays a pivotal role in maintaining the integrity of alveolar epithelial cells and microvascular structures, thereby impeding the infiltration of inflammatory cells (Jia et al., 2021b). Regrettably, the present investigation revealed that varying dosages of CCD failed to exhibit dose-dependent modulation of Nrf2/HO-1 pathway activation. Several scholars also posit that this phenomenon may be attributable to the intricate interplay between Nrf2/HO-1 pathway activation and the specific dosage range, asserting that the pathway’s protective efficacy is confined to a particular dosage spectrum (Jia et al., 2021a).

Currently, evidence does not strongly support the role of CCD in ameliorating the symptoms of cold-induced fever through an increase in the abundance of *L. murinus*. However, the experimental findings of this study indicate that CCD does modulate the abundance of *L. murinus*, which demonstrates an upward trend. Furthermore, mice exhibit antipyretic effects after oral administration of the *L. murinus* strain, which is known to mediate anti-inflammatory effects in calorie-restricted mice (Pan et al., 2018). The antipyretic mechanisms of *L. murinus* may be closely linked to the regulation of internal energy metabolism, thereby altering the inflammatory marker levels within the body and resulting in antipyretic effects. While the authors identified the associations between *L. reuteri*, *L. johnsonii*, and body temperature, individual validations or investigations were not performed for the relevant mechanistic pathways. Studies have established the role of *L. johnsonii* in effectively inhibiting the production of LPS by commensal *E. coli* and suppressing the activation of NF-κB (Gosmann et al., 2017). *L. reuteri* was found to suppress nuclear translocation of NF-κB, thereby inhibiting the binding of NF-κB to DNA or its interaction with the basal transcription machinery (Ma et al., 2004). *L. reuteri* and *L. johnsonii* may thus exert antipyretic effects by antagonizing the inflammatory processes. Intriguingly, *L. reuteri* enhances the functionality of the intestinal barrier in rats experiencing acute liver failure via the Nrf-2/HO-1 signaling pathway. Reports indicate that treatment with *L. reuteri* upregulates the expressions of Nrf-2 and HO-1 in the intestine, mitigating intestinal damage and exerting antioxidative effects (Zhou et al., 2022). Recent investigations have elucidated that the ability of *L. reuteri* to regulate redox dynamics is intricately linked with its metabolic derivative reuterin, which has been shown to exert inhibitory effects on the proliferation of colorectal cancer cells through modulation of the intracellular redox equilibrium (Bell et al., 2022). CCD may potentiate the abundance of *L. reuteri*, thereby activating the Nrf2/HO-1 pathway to modulate cold-induced inflammation and oxidative stress responses.

## 5 Conclusion

In summary, CCD treatment effectively reduces fever and improves wind-cold symptoms in mice. The underlying mechanism of action involves an increase in the abundance of *L. murinus* in the gut and regulation of the Nrf2/HO-1 signaling pathway.

## Data Availability

The 16S rRNA sequencing datasets presented in this study can be found in the NCBI database via the link https://www.ncbi.nlm.nih.gov/sra/?term=PRJNA1104442, accession number is PRJNA1104442. Other data has been aggregated into the Supplementary Material.

## Abbreviations

CCD, Cong-Chi decoction; SSP, Sojae Semen Praeparatum; NIH, National Institutes of Health; TNF-α, tumor necrosis factor α; IL-6, interleukin 6; IL-1β, interleukin-1β; cAMP, cyclic adenosine monophosphate; PGE2, prostaglandinE2; LPS, lipopolysaccharide; NSAID, non-steroidal anti-inflammatory drug; Nrf2, nuclear factor erythroid2-related factor 2; HO-1, heme oxygenase 1; WBCs, white blood cells; Lym, lymphocytes; H&E, hematoxylin and eosin; ELISA, enzyme-linked immunosorbent assay; qRT-PCR, quantitative real-time polymerase chain reaction; PVDF, polyvinylidene fluoride; HRP, horseradish peroxidase; LDA, linear discriminant analysis; Lactobacillus murinus, L. murinus; Lactobacillus johnsonii, L. johnsonii; Lactobacillus reuteri, L. reuteri.
