# Supplementary figures and images for: Decoction derived from Allium ascalonicum L. bulbs and Sojae Semen Praeparatum alleviates wind-cold-type common cold via Nrf2/HO-1 pathway and modulation of Lactobacillus murinus level
Source: Front Pharmacol. 2024 May 13;15:1364328. doi: 10.3389/fphar.2024.1364328 (PMC11129017; doi:10.3389/fphar.2024.1364328)

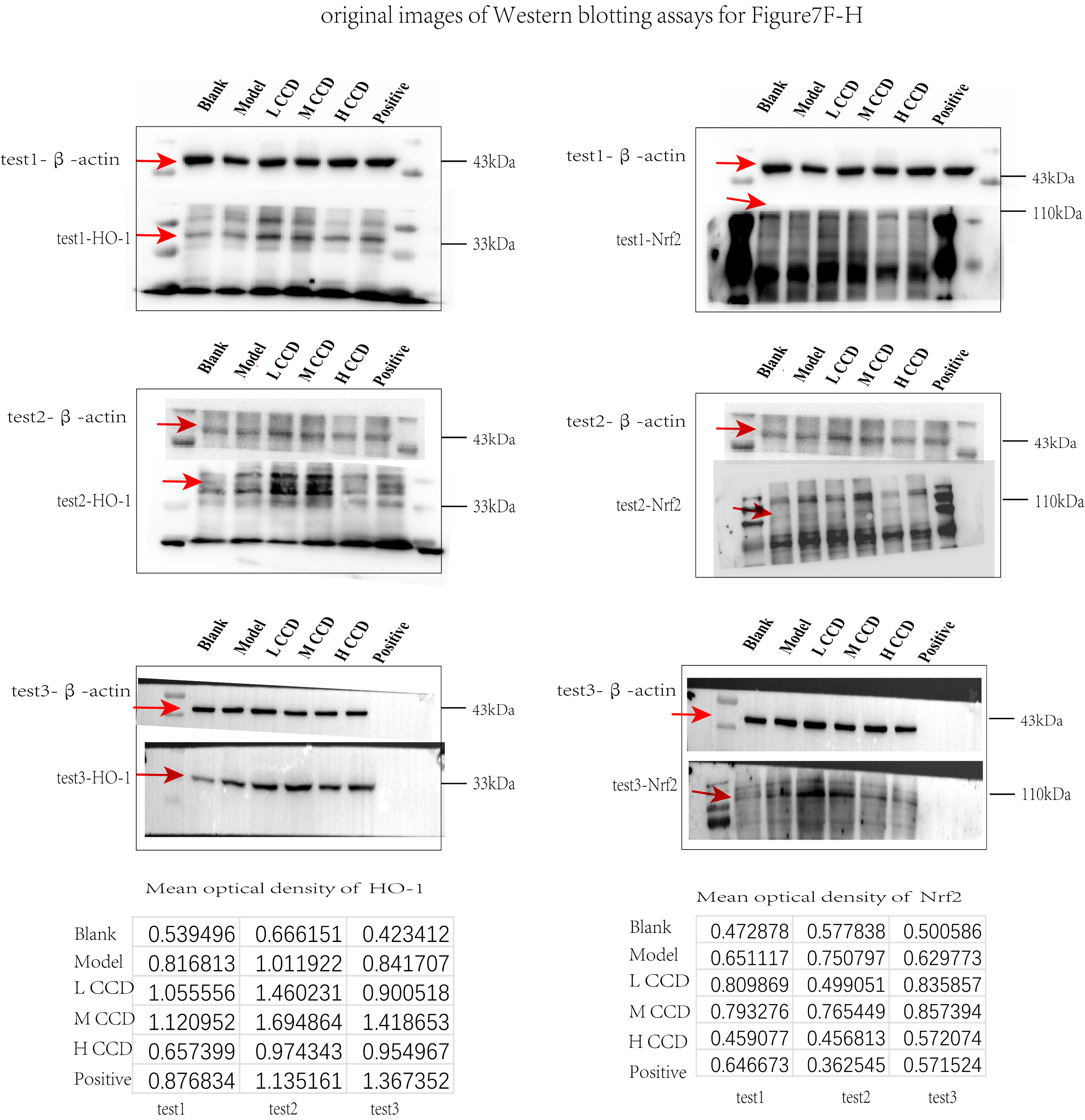

Supplement: Supplementary file 1 [file Image1.TIF]

**Daidzein**


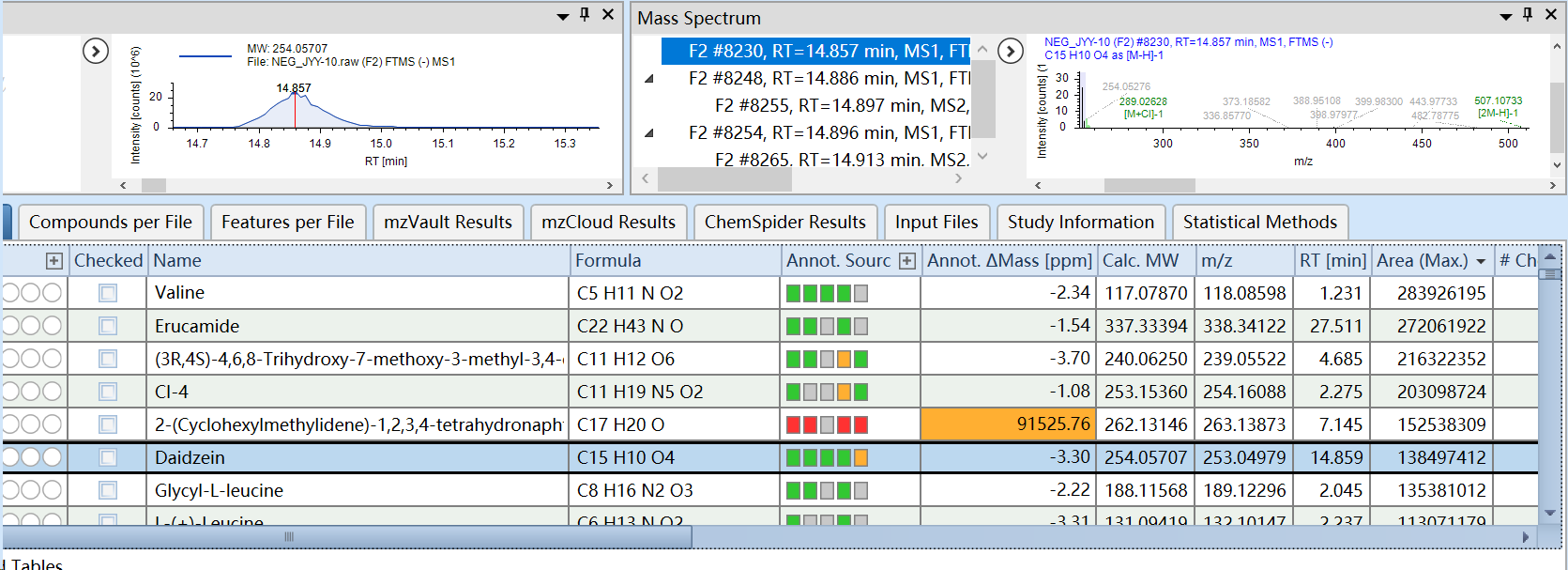


**Genistein**


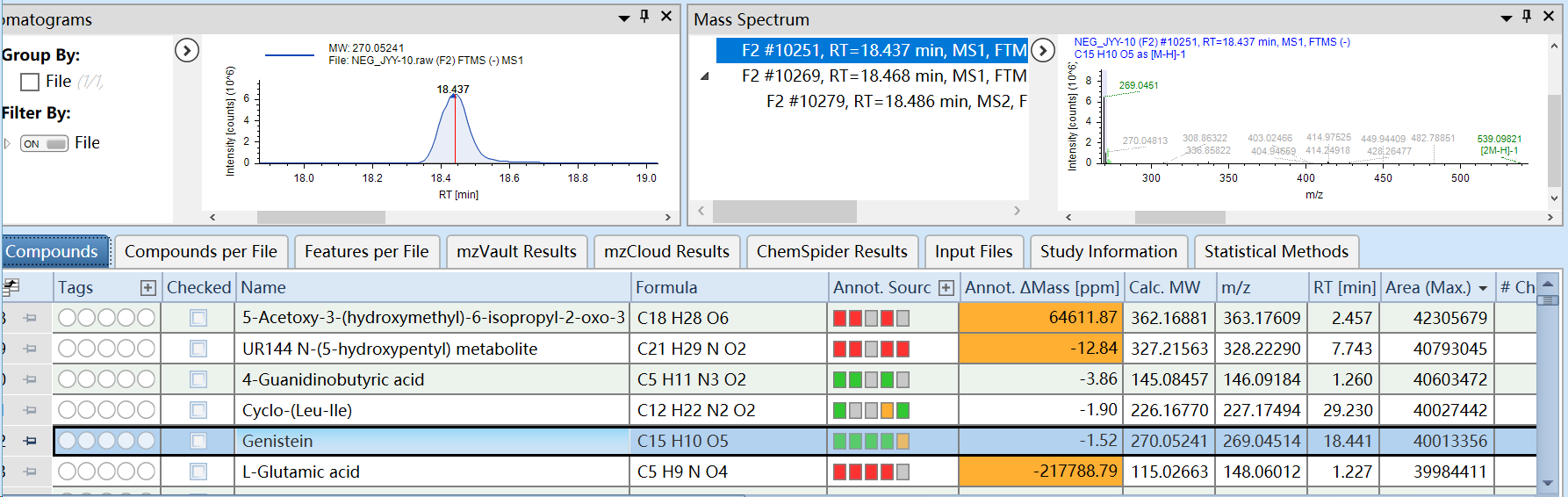


**Glycitein**


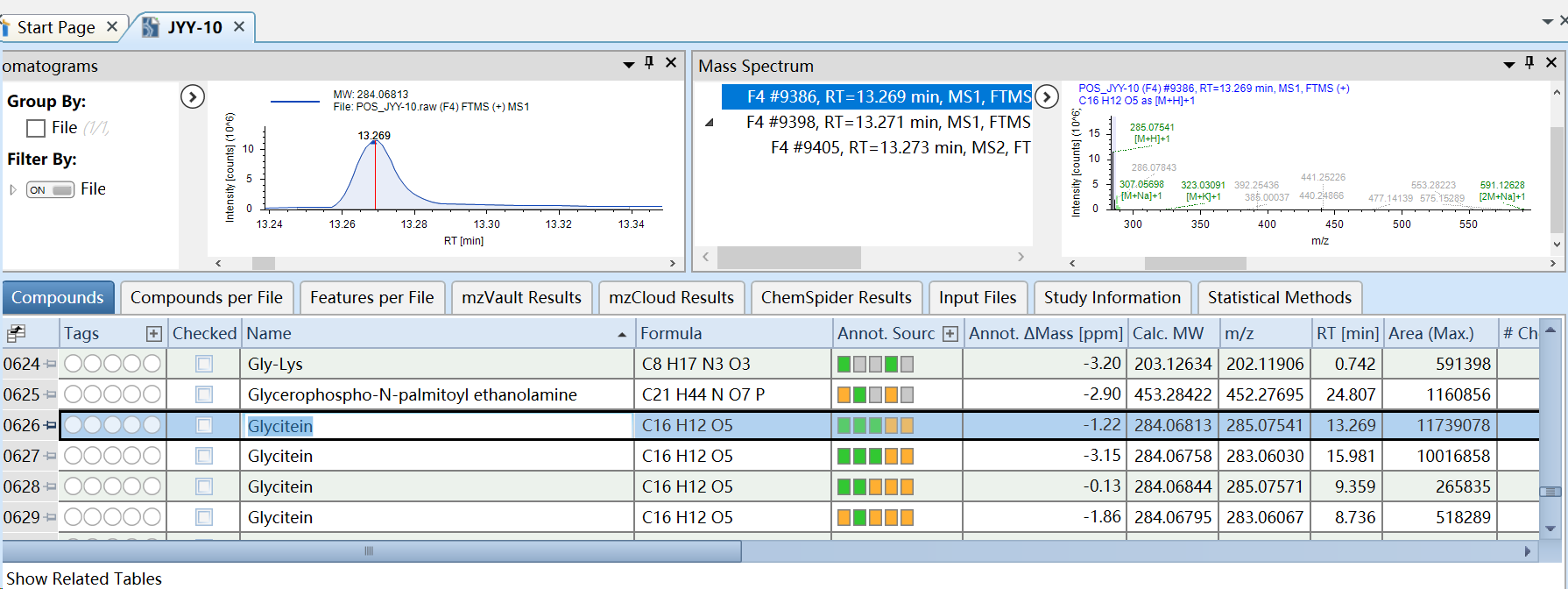


**S-Methyl-L-cysteine-S-oxide**


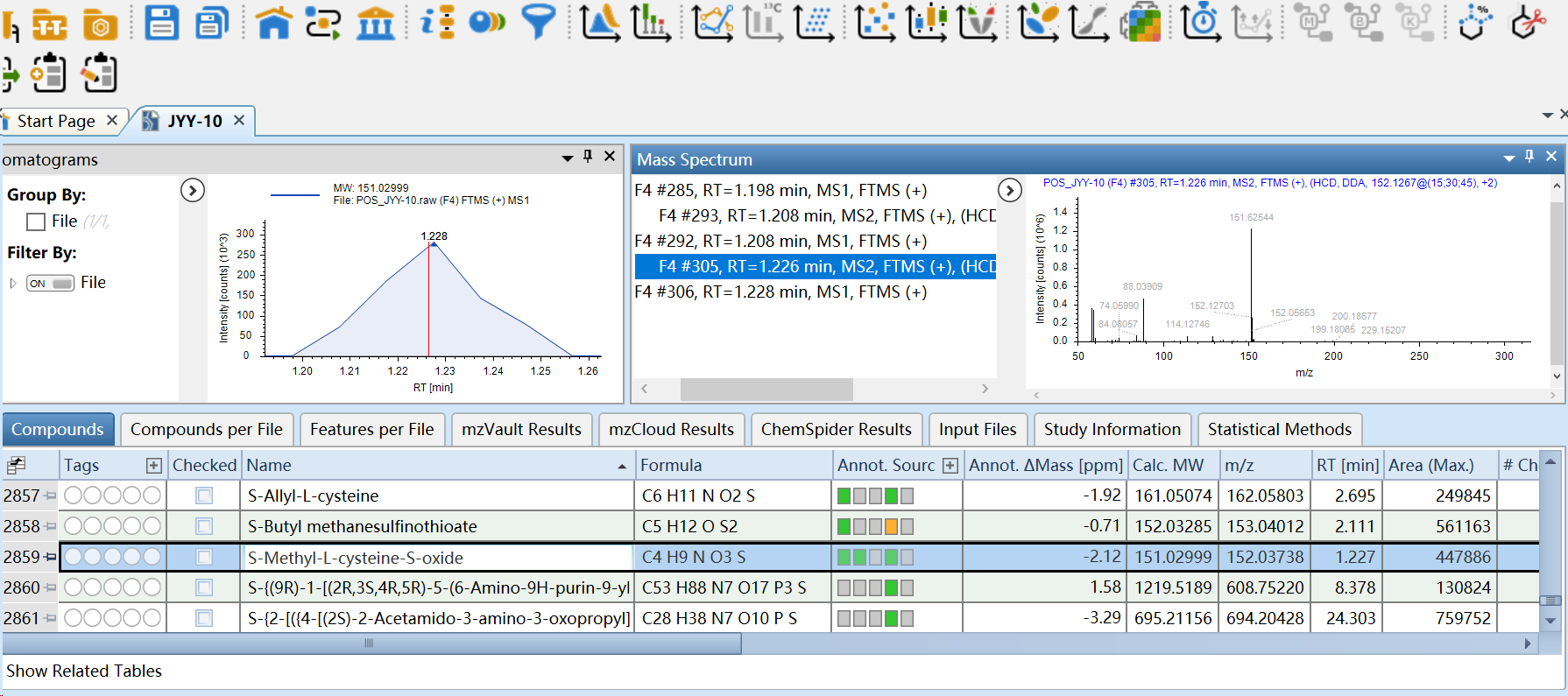

Supplement: Supplementary file 3 [file DataSheet1.docx]
